# Applicability, Safety, and Efficiency of Salpingectomy versus Electrocoagulation and Laparoscopic Tubal Section in Ambulatory

**DOI:** 10.1055/s-0042-1755243

**Published:** 2022-08-29

**Authors:** Joana Margarida Araújo Pereira, Vera Filipa Batista Trocado, Sandra Marina Sousa Gomes, Mariana Carlos-Alves, Agostinho Carvalho, Paula Alexandra Pinheiro

**Affiliations:** 1 Serviço de Ginecologia e Obstetrícia, Unidade Local de Saúde do Alto Minho, Viana do Castelo, Portugal; 2 Instituto de Investigação em Ciências a Vida e Saúde, Escola de Medicina, Universidade do Minho, Portugal

**Keywords:** female, sterilization, salpingectomy, laparoscopic surgery, ovarian cancer, mulher, esterilização, salpingectomia, cirurgia laparoscópica, câncer do ovário

## Abstract

**Objective** Female sterilization is a surgical procedure that aims women to permanently stop the use of conception. The benefits, risks and cost-effectiveness are important issues. The purpose of this study was comparing the applicability, complications and efficacy of salpingectomy versus electrocoagulation and tubal occlusion by laparoscopy in the Ambulatory Surgery Unit.

**Methods** We performed a retrospective and observational study that included women undergoing laparoscopic sterilization procedures at our Ambulatory Surgery Unit, during three years. Statistical analysis was performed using SPSS, applying the Fisher exact test, the Mann-Whitney test, and Linear Regression.

**Results** Two hundred and twenty-one laparoscopic surgical procedures were performed, including 79 (35.7%) bilateral total salpingectomies and 142 (64.3%) electrocoagulation and bilateral tubal occlusion procedures. The majority of the procedures were performed by a resident ( *n* = 162; 73.3%), with 40% ( *n* = 33) of salpingectomies. The surgical time, independently the type of surgeon, was significantly shorter in the tubal occlusion (42.2 vs. 52.7 min, *p* < 0.001). Safety and efficacy endpoints were not significantly different between the two groups, with a case of pregnancy in tubal occlusion group.

**Conclusion** Salpingectomy is a safe and effective alternative comparing with electrocoagulation and tubal occlusion.

## Resumo

**Objetivo** A esterilização feminina é um procedimento cirúrgico que auxilia as mulheres na paragem permanente de utilização de métodos contraceptivos. Os objetivos desde estudo foram comparar a aplicabilidade, complicações e eficácia da salpingectomia vs. eletrocoagulação e secção tubar na esterilização feminina em regime de ambulatório.

**Métodos** Realizou-se um estudo retrospectivo e observacional que incluiu mulheres submetidas a procedimentos de esterilização por laparoscopia no Serviço de Cirurgia de Ambulatório da ULSAM, durante três anos. A análise estatística foi realizada com recurso ao SPSS, aplicando o teste exato de Fisher, o teste de Mann-Whitney e Regressão Linear.

**Resultados** Foram realizados 221 procedimentos cirúrgicos por laparoscopia, incluindo 79 (35,7%) salpingectomias totais bilaterais e 142 (64,3%) procedimentos por eletrocoagulação e secção tubar bilateral. A maioria dos procedimentos foram realizados por um interno de formação específica ( *n* = 162; 73,3%), com 40% ( *n* = 33) de salpingectomias. O tempo operatório foi significativamente inferior no grupo da eletrocoagulação (42,2 vs. 52,7 min, *p* < 0,001). Em relação à segurança e à eficácia não se observaram diferenças estatisticamente significativas entre os dois grupos, com um caso de gravidez após eletrocoagulação e secção tubar.

**Conclusão** A salpingectomia é uma alternativa segura e com alta taxa de eficácia quando comparada com eletrocoagulação e secção tubar.

## Introduction

Female sterilization includes a number of different procedures and techniques that provide permanent contraception for women. Methods of female sterilization include tubal occlusion, partial salpingectomy, and total salpingectomy. The only indication for female permanent contraception is patients' preference to have a permanent method of contraception for pregnancy prevention. 1

Female permanent contraception can be performed using several different procedures and techniques that prevent pregnancy by occluding or removing the fallopian tubes. 1 Laparoscopy is the most common surgical approach. 1 Tubal sterilization by cutting ant tying the fallopian tube, or using electric current, clips or rings, is an effective method of contraception. 2 Salpingectomy is the complete removal of the fallopian tubes.

Major complications are considered to be the need for any surgery such as unintentional laparotomy, perforate viscera repair, rupture large vessels, loss of blood greater than 500 ml, blood transfusion needs, febrile morbidity, or potentially fatal events. Minor complications include uterine lesions, small vessel injuries, paralytic ileus, wound dehiscence, and urinary tract infections. 3

The overall complication rate of laparoscopic sterilization is low. The rate of major complications observed in a prospective cohort study was 1.6%, and performing an unintentional laparotomy and rehospitalization were the most reported complications. The rate of minor complications was 0.26%, and uterine injury was the most frequent complication. 3 Estimates of failure rates for tubal section were less than 5 pregnancies per 1000 procedures in the first-year poststerilization. 2

The American College of Obstetricians and Gynecologists (ACOG) and the American Cancer Association (ACS) recommend opportunistic salpingectomy for primary prevention of epithelial carcinoma of the fallopian tube, ovary, or peritoneum in a woman undergoing pelvic surgery for another indication, including desire of permanent contraception. 4 5 According some authors, women who had undergone a tubal ligation or a bilateral salpingectomy had a reduction risk to develop ovarian cancer, 24 and 65%, respectively, when compared with women who did not have performed this procedure. Moreover, salpingectomy offers a 100% efficacy compared with other methods. 4 6

Because salpingectomy has been associated with a concern over its potentially damaging effect on the ovarian reserve, during many years procedures like tubal occlusion were preferred. However, recent studies showed that bilateral salpingectomy did not cause any decline in serum Anti-Müllerian Hormone concentration, despite the expected increase in damage to the ovarian blood supply. 4 7

## Methods

This is a retrospective cohort study of women who underwent laparoscopic surgical sterilization, performed at the Ambulatory Surgical Center in the Alto Minho Local Healthcare Unit (ULSAM), Viana do Castelo, Portugal, during January of 2016 and December of 2018. Inclusion criteria were all women undergoing a laparoscopic surgical sterilization during this time. The exclusion criteria were performing more than one surgical procedure for sterilization, and loss of follow-up.

This study was conducted with the approval of the Ethics Committee of ULSAM, with no need for Institutional Review Board approval.

Patients' charts were reviewed to obtain demographic information (age, body mass index, smoking status, and parity), presence of medical and surgical comorbidities (endometriosis, inflammatory pelvic disease, and abdominal or pelvic surgery), level of surgeon (resident vs. attending), type of procedure, number of abdominal ports, surgical time, acute, short, and long-term complications, and effectiveness of procedure. A number was assigned to each patient, in order to maintain data confidentiality.

The laparoscopic approach in ULSAM is the salpingectomy using the bipolar Maryland forceps for cauterization, followed by scissors for transection. Patients who underwent other procedures were excluded. Surgical time was defined from incision to closure. Acute complications (during the procedure or prior to leaving the ambulatory center) included hemorrhage, pain, needs of hospitalization, uterine perforation, and complications related to insufflation of gas. Short-term complications (within 24 hours postdischarge) were assessed through telephone contact, namely pain, fever, suture bleeding, nausea and vomiting, and normal intestinal function. Long-term complications were assessed during standard of care, postoperative visits, and urgency care visits, where infection, pain requiring additional visits, and readmission to the hospital were searched.

Continuous variables were described using median and standard deviation, and categorical variables using frequencies. Continuous variables were compared between groups using the Mann-Withney test, and dichotomous variables using the Fisher exact test or the Chi-square test, as appropriate. A linear regression and a logistical regression were performed to evaluate possible confounding or adjustments factors. The odds ratio (OR) and 95% confidence interval (CI) were calculated. Statistical analyses were performed with the the Statistical Package Social Sciences (SPSS, IBM Corp., Armonk, NY, USA) version 23. Statistical significance was defined as *p* -values < 0.05.

## Results

The study population includes all women undergoing laparoscopic sterilization in the Ambulatory Surgery Unit ( *n* = 221). Procedures included 79 (35.7%) salpingectomies and 142 (64.3%) tubal occlusions. No significant differences were verified between the median of age (38 vs. 38 years, *p* = 0.86) and median of body mass index (BMI) of the two groups (26.30 vs. 26.23 kg/m ^2^ , *p* = 0.50). In this sample, only one woman had a documented history of endometriosis. The most used contraceptive method before surgery was combined pill ( *n* = 81; 36.7%) and the least was natural family planning ( *n* = 3; 1,4%). In the study population, 34.2% of women ( *n* = 75) had a history of abdominal or pelvic surgery. Patient characteristics for the 221 women are presented in Table 1 .

**Table 1:** Demographics and medical history of women submitted to sterilization from January of 2016 to December of 2018

	All	Electrocoagulation and tubal section	Salpingectomy	*p* -value
	Median (quartiles) / (frequencies, %)	*n* = 142	*n* = 79	
Age (years)	37.89 (27–46%)	37.7	38.1	0.625
BMI (kg/m ^2^ )	25.96 (17.2–40.2%)	25.8	26.1	0.609
**Gravidity**				
1	32 (14.5%)	17	15	
2	94 (42.5%)	66	28	
≥ 3	95 (43%)	59	36	
**Parity**				
1	41 (18.6%)	24	17	
2	117 (52.9%)	83	34	
≥ 3	63 (28.5%)	35	28	
Tobacco use	19 (8.6%)	13	6	0.449
**Medical conditions**				
Diabetes	2 (1%)	0	2	0.296
Hypertension	20 (9%)	14	6	0.394
Human Immunodeficiency Virus	1 (0.5%)	0	1	0.276
Thyroid Pathology	13 (5.9%)	7	6	0.291
Thrombosis history	8 (3.6%)	6	2	0.421
Gynecology history				
Endometriosis	1 (0.5%)	0	1	0.354
Inflammatory pelvic disease	3 (1.4%)	1	2	0.292
Prior abdominal or pelvic surgery	75 (33.9%)	49	26	0.88

**Abbreviation:** BMI, body mass index.

In this study, 73.3% ( *n* = 162) of procedures were performed by resident physicians, where 40% ( *n* = 33) of those were salpingectomies. Average surgical times were 10.5 minutes longer for salpingectomy compared to tubal occlusion method (52.7 vs. 42.2 min, respectively; *p* < 0.001; 95% CI = 6.5–13.6), after control for BMI, prior abdominal or pelvic surgery and procedure realized by a resident ( Table 2 ).

**Table 2:** Characteristics of procedure of groups with respective *p* -values

	Electrocoagulation and tubal section	Salpingectomy	*p* -value
	*n* = 142	*n* = 79	
**Resident surgeon**	107 (75.3%)	55 (69.6%)	0.35
**Surgery time (min)**	42.2	52.7	**< 0.001**
**Number of ports**	2.08	3.01	**0.033**
**Acute complications**	4 (2.8%)	0 (0%)	–
**Needs of hospitalization**	2 (0.9%)	0 (0%)	–
**Long-term complications**	4 (2.8%)	2 (2.5%)	0.64
**Efficacy**	141 (99.3%)	79 (100%)	0.64

**Notes:***p-* values in bold were statically significant.

Regarding complications, 4 acute complications occurred in the tubal occlusion group (2 mesosalpinx hemorrhages that required an overnight in the Gynecology Department, 1 subcutaneous emphysema, and 1 uterus perforation), with no registered acute complications in the salpingectomy group (2.8 vs. 0%). Furthermore, 24 hours after surgery, the short-term complications were assessed by telephone contact ( *n* = 175); this group included 68 (38.9%) salpingectomy and 107 (61.1%) tubal occlusion procedures. There were no differences between short-term complications in both groups ( Table 3 ).

**Table 3:** Postoperative outcomes according to type of procedure

	Electrocoagulation and tubal section	Salpingectomy	*p* -value
	*n* = 107	*n* = 68	
Fever	0 (0%)	0 (0%)	–
Nausea and vomiting	0 (0%)	1 (1.5%)	–
Suture bleeding	3 (2.8%)	0 (0%)	–
Normal intestinal function	45 (42.1%)	22 (32.4%)	0.198
Pain (> 5) ^*^	3 (2.8%)	4 (6%)	0.295

**Notes:** * Pain was rated on an increasing analog scale from 1 to 9.

When evaluating postoperative pain, no significant differences were registered between both groups, even after controlling for number of ports, surgery time, level of the surgeon, and prior abdominal or pelvic surgeries. Long-term complications for the salpingectomy group included 1 wound infection requiring oral antibiotics and 1 wound dehiscence (without need for reintervention). In the tubal occlusion group, it was registered 2 cases of wound infection, and 1 of wound dehiscence (without need of reintervention), but without significant differences between the groups (2.5% vs. 2.8%, *p* = 0.64). Procedure efficacy didn't show significant differences between the salpingectomy and tubal occlusion groups (100 vs. 99.3%, respectively; *p* = 0.64) with 1 pregnancy registered in the tubal occlusion group.

## Discussion

This study suggests that salpingectomy in sterilized procedure is a safe procedure. No safety concerns were found (acute, short or long-term complications) comparing laparoscopic salpingectomy and tubal occlusion for women sterilization, as suggested in previous studies. 4 8 9 This study showed a higher rate of acute complications (0 vs. 2.8%) with an increase of hospitalization (0 vs. 0.9%) in the tubal occlusion group compared with the salpingectomy group; however, there was no statistically significant difference between groups. Although the literature describes a higher risk of surgical complications in women with diabetes, obesity, and previous abdominal or pelvic surgeries, this was not confirmed in our sample.

The surgical time of salpingectomy is slightly longer than the tubal occlusion method, without significant differences of complications rates. 4 The average surgical time was 10.5 min higher in the salpingectomy group, when compared with the tubal occlusion group. These results support the findings of Westberg et al. 8 (additional time of 6 minutes) and Hanley et al. 10 (additional time of 16 min). After control for BMI, prior abdominal or pelvic surgeries, and procedure performed by a resident physician, the time of surgery was defined by the type of procedure chosen. This data is in accordance with Wong et al., 11 who showed that salpingectomy can be effective regardless of the surgeon's level.

In this study, the efficacy of tubal occlusion was 99.3% (with 1 case of pregnancy during the first year after the procedure, 7 per 1,000 procedures) and 100% in salpingectomy. According to the Collaborative Review of Sterilization (CREST) study, the cumulative 10-year probability of pregnancy following tubal ligation was 18.5 per 1,000 procedures, and 7.5 per 1,000 with unipolar coagulation and postpartum partial salpingectomy. 11 Young age at the time of sterilization has been determined to be the main predictor of failure. 12

The ovarian function was not assessed in this study; however, in a systematic review, Mohamed et al. 7 showed that radical salpingectomy doesn't seem to interfere with the ovarian reserve in the short term. However, the long-term effect remains uncertain, predicting a possible concomitant damage to the ovarian blood supply.

According to ACOG and ACS, salpingectomy offers the opportunity to significantly decrease the risk of ovarian cancer. 4 5 Women with the BRCA-1 mutation are also found to have a risk reduction of ovarian cancer. 9 Wong et al. 11 showed that for sterilization, salpingectomy is more costly than tubal occlusion, but more effective. Kwon et al., 13 using a statistic model designed to determine cost-effectiveness of opportunistic salpingectomy, showed that salpingectomy had to provide a relative increase in risk reduction of 25% over tubal ligation, and according to the authors' model, there is a relative risk reduction of 29.2% in ovarian cancer cases with the use of salpingectomy versus tubal ligation.

Some limitations of this study should be noted, such as its retrospective nature, the relatively small sample size, the fact that some patients could be followed up and/or admitted in a different hospital, and the short follow-up time to assess procedure efficacy. However, this is an original study, and there are few studies in this area which study this important question.

## Conclusion

Our study shows that the salpingectomy procedure is possible and safe at the Ambulatory Surgery Unit, preventing tubal torsion surgery, hydrosalpinx, or ectopic pregnancy. An improvement of surgical time could be achieved with more training and experience of surgeons in laparoscopy and salpingectomy procedures.
